# Cloud–Edge–End Collaborative Federated Learning: Enhancing Model Accuracy and Privacy in Non-IID Environments

**DOI:** 10.3390/s24248028

**Published:** 2024-12-16

**Authors:** Ling Li, Lidong Zhu, Weibang Li

**Affiliations:** 1 National Key Laboratory of Wireless Communications, University of Electronic Science and Technology of China, Chengdu 611731, China; 202111220609@std.uestc.edu.cn; 2 School of Computer Science and Engineering, Southwest Minzu University, Chengdu 610041, China; 21700142@swun.edu.cn

**Keywords:** cloud–edge–end architecture, federated learning, privacy preserving, edge computing

## Abstract

Cloud–edge–end computing architecture is crucial for large-scale edge data processing and analysis. However, the diversity of terminal nodes and task complexity in this architecture often result in non-independent and identically distributed (non-IID) data, making it challenging to balance data heterogeneity and privacy protection. To address this, we propose a privacy-preserving federated learning method based on cloud–edge–end collaboration. Our method fully considers the three-tier architecture of cloud–edge–end systems and the non-IID nature of terminal node data. It enhances model accuracy while protecting the privacy of terminal node data. The proposed method groups terminal nodes based on the similarity of their data distributions and constructs edge subnetworks for training in collaboration with edge nodes, thereby mitigating the negative impact of non-IID data. Furthermore, we enhance WGAN-GP with attention mechanism to generate balanced synthetic data while preserving key patterns from original datasets, reducing the adverse effects of non-IID data on global model accuracy while preserving data privacy. In addition, we introduce data resampling and loss function weighting strategies to mitigate model bias caused by imbalanced data distribution. Experimental results on real-world datasets demonstrate that our proposed method significantly outperforms existing approaches in terms of model accuracy, F1-score, and other metrics.

## 1. Introduction

### 1.1. Background

With the rapid development of Internet of Things (IoT) and 5G technologies, cloud–edge–end collaborative computing architecture has become a crucial paradigm for processing and analyzing large-scale edge data. In this architecture, data are typically distributed across multiple terminal nodes and processed hierarchically through edge nodes and cloud servers. This architecture combines the powerful computing capabilities of cloud computing, the low-latency characteristics of edge computing, and the ubiquity of terminal devices. It is particularly important in application scenarios that require real-time data processing and decision-making, such as autonomous driving, smart cities, and industrial IoT, while also providing new possibilities for various application scenarios [1]. However, in this distributed environment, data privacy protection and model performance optimization have become two conflicting yet equally important objectives.

In recent years, edge computing, as a new computing model that deploys computing and storage resources at the network edge, has gradually become a focus of attention in academia and industry [2]. It can process data near the data source, thereby reducing network latency, improving real-time performance, and reducing bandwidth pressure. Meanwhile, federated learning, as a distributed machine learning paradigm, allows multiple participants to jointly train models without directly sharing raw data. Terminal devices only need to perform model training on local data and share model updates to a central aggregator, providing a new solution for data privacy protection [3].

However, in practical applications, client data often exhibits non-independent and identically distributed (non-IID) characteristics, which poses significant challenges to traditional machine learning algorithms and federated learning frameworks [4]. Non-IID data distribution may lead to bias in model updates, slow down model convergence, and reduce the overall performance of the global model. Therefore, how to effectively handle non-IID data while protecting client data privacy and improving model accuracy has become a key issue in current research.

### 1.2. Motivation and Contributions

In cloud–edge–end scenarios, the non-IID nature of terminal node data exacerbates the difficulty of improving federated learning model accuracy while ensuring data privacy. Data heterogeneity may lead to suboptimal performance of the global model and unbalanced effects on different edge nodes. Furthermore, how to effectively protect client data privacy during the training and aggregation process and prevent sensitive information leakage remains an important research topic.

The main motivations for this study stem from the following aspects:

Increasing urgency of data privacy protection: With the implementation of data security regulations (such as GDPR, CCPA) and the increasing awareness of user privacy, how to protect user privacy while utilizing distributed data for machine learning has become an urgent problem to be solved [5].

Challenges brought by non-IID data: In practical applications, client data usually exhibits non-IID characteristics, which severely affects the performance of traditional federated learning algorithms. How to effectively handle non-IID data has become the key to improving model accuracy [6].

Need for integration of edge computing and federated learning: Edge computing provides a new computing paradigm for federated learning, but how to effectively implement federated learning under the limited resources of edge devices still faces many challenges [7].

Trade-off between model accuracy and privacy protection: Improving model accuracy while protecting data privacy are often conflicting goals. How to find the optimal balance between the two is one of the core motivations of this research [8].

Urgent needs in practical application scenarios: In fields such as smart healthcare, financial technology, and intelligent transportation, there is an urgent need to build high-accuracy machine learning models using distributed data while protecting data privacy [9].

This research aims to address the privacy protection and model accuracy optimization issues in cloud–edge–end scenarios based on edge computing and federated learning. The main contributions are as follows:

Subnetwork Partitioning Strategy: We propose a novel subnetwork partitioning method based on data similarity, aimed at improving the accuracy of local models within each subnetwork and, consequently, enhancing the performance of the global model.

Privacy protection technology: For non-IID data scenarios, we combine GAN with differential privacy (DP) techniques. By generating datasets for training at terminal nodes, we effectively mitigate the negative impact of non-IID data on model training, ensuring effective protection of terminal node data privacy while improving model accuracy.

Evaluation and validation: We conduct extensive experiments using real datasets in simulated cloud–edge–end architectures to verify the effectiveness of the proposed method in improving model performance and protecting data privacy under non-IID data conditions.

## 2. Related Work

In cloud–edge–end architectures, research on the combination of edge computing and federated learning has garnered increasing attention, especially in addressing how to effectively protect client data privacy while improving model accuracy in non-IID data scenarios. This section will elaborate on related work in four aspects: cloud–edge–end architecture, edge computing, federated learning, and privacy protection.

### 2.1. Cloud–Edge–End Architecture

The cloud–edge–end three-layer architecture is a distributed computing model that distributes computing, storage, and network resources across cloud, edge nodes, and terminal devices [10]. In this architecture, data collection, processing, and storage tasks are processed in layers according to requirements. The cloud is typically responsible for complex computational tasks and large-scale data storage, edge nodes handle near real-time local processing and response, and terminal devices perform data collection and preliminary processing. The advantage of this architecture lies in reducing network latency, lowering cloud load, and improving system response speed by pushing computing resources to the edge.

Cloud–edge–end collaboration is the core concept of the cloud–edge–end architecture, emphasizing the cooperation and complementarity between the cloud, edge, and end layers. Zhang et al. [11] proposed a cloud–edge–end collaborative architecture supporting AIGC for autonomous driving, building mutually supportive AIGC and network systems for autonomous driving, and utilizing AIGC to assist system design and resource management. Fang et al. [12] proposed a novel Gold sequence-based secure network coding (GS-SNC) scheme for the security lightweight problem of Cloud–Edge–Terminal Collaborative AI-empowered Internet of Things (CETC-AIoT) data. The scheme introduces Gold sequences to generate pseudo-random sequences for scrambling and descrambling original information and constructs pre-coding matrices for encoding and decoding scrambled information, enhancing security while reducing computational complexity and space overhead. Addressing the complexity of the Virtual Network Embedding (VNE) problem and network security requirements, Zhang et al. [13] designed a resource allocation mechanism based on VNEVNE (DRLS-VNE), which effectively improves the performance and security of DRLS-VNE by establishing a multi-dimensional heterogeneous network model and introducing a dynamic trust evaluation mechanism. Yu et al. [14] adopted a game theory approach and proposed a three-layer 5G distributed cloud–edge–end network model containing an attack-defense game model between DoS attackers and SDN defenders for the attack-defense interaction problem in diverse services of Mobile Edge Computing (MEC).

### 2.2. Edge Computing

Edge computing, as a key component of cloud–edge–end architecture, has received widespread attention in recent years. Edge computing processes data near its source, reducing latency and bandwidth consumption while supporting heterogeneous device collaboration and ensuring data privacy in localized environments.

Edge intelligence is the combination of edge computing and artificial intelligence, aiming to push AI capabilities to edge devices. Huang et al. [15] proposed a multi-cell collaborative sensing ISAC scheme for edge intelligence, which offloads sensing data to edge servers with sufficient computing power for model training through a multi-cell collaborative method, showing significant advantages in reducing the overall power consumption of ISAC stations. Zeng et al. [16] introduced a model-free Deep Reinforcement Learning (DRL) method to effectively manage resources at the network edge, addressing the limitations of traditional model-based resource management methods in edge computing. This method can adapt well to network dynamics without any prior knowledge. Addressing the key security issue of how to accurately and efficiently verify the integrity of edge data in edge computing environments, Cui et al. [17] proposed a scheme called ICL-EDI for EDI checking and corruption localization to solve the Edge Data Integrity (EDI) problem. This scheme allows service providers to accurately and effectively check data integrity and locate corrupted edge data cached on multiple edge servers. To address the privacy leakage issues arising during the offloading process in Mobile Edge Computing (MEC), Wu et al. [18] proposed a privacy-preserving offloading scheme considering multiple access points based on stochastic game theory and introduced a Joint Optimal and Privacy-Preserving DRL algorithm (JODRL-PP) to achieve the optimal offloading scheme for the system.

Resource constraints and data heterogeneity are key challenges in edge computing. Especially in cases where terminal device data exhibits non-IID characteristics, finding methods to efficiently utilize edge nodes for data processing and model training has become a research hotspot.

### 2.3. Federated Learning with Non-IID Data

Federated learning (FL) [19] is a distributed machine learning method that allows multiple parties (such as terminal devices or edge nodes) to jointly train a global model without sharing their local data. This method enhances data privacy by avoiding centralized data processing through local model updates. Federated learning has been applied in various fields such as mobile devices, healthcare, and financial services.

Non-independent and identically distributed (non-IID) data are one of the main challenges faced by federated learning. Specifically, non-IID data can lead to large biases in local model updates, thereby affecting the convergence and accuracy of the global model. Therefore, optimizing federated learning algorithms for non-IID data distributions has become a current research focus.

Addressing the problem of model performance bias and imbalance in federated learning due to the presence of non-independent and homogeneous distribution of data, Lu et al. [20] argue that the non-independent and identically distributed (non-IID) nature of data in federated learning can lead to divergent parameter values across local models, resulting in inconsistent updates during aggregation and conflicts in global model parameters. This phenomenon prevents the global model from converging to an optimal state, or significantly extends the time required for convergence. To address the challenge of slow training progress caused by heterogeneous data distributions across clients in federated learning, Lee et al. [21] proposed a novel algorithm called DOCS. This approach identifies client clusters with data distributions closely resembling IID characteristics and employs multi-armed bandit techniques to select clusters with the fastest convergence rates, thereby enhancing the overall efficiency of federated learning. Younis et al. [22] proposed a new method called FLY-SMOTE to deal with non-IID data in federated learning by rebalancing the data in different non-independent and identically distributed scenarios in order to solve the problem of unbalanced fault data in equipment fault detection.

To address the challenges of statistical heterogeneity in federated learning, Zhang et al. [23] introduced a DRL-based approach for adaptive control of local model training and global aggregation phases, better accommodating non-IID data characteristics. Gao et al. [24] proposed a federated learning algorithm with local drift decoupling and correction capabilities, introducing auxiliary local drift variables to track disparities between local and global model parameters. While this method enhances model federation performance, it carries a risk of overfitting. Liang et al. [25] introduced Random Sampling Consensus FL (RSCFed) which employs stochastic double sampling to extract multiple sub-consensus models from each client for aggregation, effectively eliminating consensus bias caused by non-uniform reliability models.

Addressing stability challenges from non-IID data in federated learning, You et al. [26] proposed Federated Gradient Scheduling (FedGS), an optimizer that relabels clients and their submitted gradients based on client label distributions. This approach reduces the unstable effects of data heterogeneity on historical gradients and improves convergence and stability by treating clients with similar label distributions as a group. Wang et al. [27] employed partial average gradient scheduling and global stochastic gradient momentum for asynchronous training with non-IID datasets in edge environments, reducing gradient update fluctuations and enhancing training stability. Zhou et al. [28] introduced Two-level Weighted K-async Federated Learning (WKAFL) which incorporates an adaptive learning rate mechanism to avoid gradient update instability and improve model accuracy.

Addressing privacy and security challenges from non-IID data in federated learning, Zhang et al. [29] proposed an Asynchronous Group-based Federated Learning (AG-FL) framework, introducing an Adaptive Rényi Differential Privacy Budget Allocation (ARB) protocol and Asynchronous Weighted Group-based Update (AWGU) algorithm. This approach adjusts privacy budgets based on device privacy sensitivity to protect local model privacy. Xiong et al. [30] introduced 2DP-FL, an algorithm that ensures model convergence and privacy protection by adding noise during both local model training and global model distribution processes. Wang et al. [31] developed a novel Byzantine-robust federated learning framework based on isolation forest principles, which identifies and excludes potentially malicious model updates from global model aggregation by reducing the number of split-isolated model updates, thereby ensuring global model security and reliability.

### 2.4. Privacy Preserving

Privacy protection has become increasingly important in the current digital age, especially in the fields of machine learning and data analysis. Federated learning, as a distributed machine learning paradigm, inherently possesses certain privacy protection characteristics. However, researchers continue to explore how to further enhance privacy protection in federated learning. Wei et al. [32] proposed a new federated learning framework based on the concept of differential privacy which adds artificial noise to parameters on the client side before aggregation, i.e., noise analysis before model aggregation FL (NbAFL). This framework can significantly improve model performance while protecting individual privacy. To address the challenge in differential privacy methods of balancing noise injection with accuracy loss, Talaei et al. [33] proposed an adaptive noise addition method for federated learning. This method determines the amount of noise to inject based on the relative importance of features. By adding more noise to less important parameters and less noise to more important parameters, it helps to effectively preserve model accuracy while protecting privacy. Byali et al. [34] proposed a federated learning framework based on secure multi-party computation which enables efficient model training without revealing individual data. Their method significantly reduces communication overhead, making large-scale federated learning possible.

Homomorphic encryption allows computations on encrypted data without the need for decryption first, which has important applications in the field of privacy protection. Menon et al. [35] proposed a single-server private information retrieval (PIR) protocol that relies on a combination of two lattice-based homomorphic encryption schemes: the Regev encryption scheme and the Gentry–Sahai–Waters encryption scheme. This protocol greatly improves the efficiency of private information retrieval. Xu et al. [36] studied the application of homomorphic encryption in edge computing and proposed a lightweight edge computing data privacy protection scheme based on blockchain and homomorphic encryption, which can effectively encrypt and transmit data.

With the increasing demand for explainability in AI systems, the explainability of privacy protection has also become a research hotspot. Li et al. [37] proposed a simple and effective Adaptive Differential Privacy (ADP) mechanism that selectively adds noise perturbations to the gradients of client models in federated learning to avoid sensitive information leakage and theoretically analyzed the impact of gradient perturbation on model explainability. Wu et al. [38] proposed a novel privacy-preserving and explainable vertical federated learning (VFL) system that not only provides robust and efficient privacy protection for various ML models but also promotes understanding of VFL model predictions through a flexible and privacy-preserving explainability framework.

Although there has been considerable research on cloud–edge–end computing, federated learning, privacy protection, and related topics, organically combining these technologies to solve practical problems still faces numerous challenges. This research will provide new solutions for privacy-preserving machine learning in cloud–edge–end scenarios, promoting the development and application of related technologies.

## 3. Cloud–Edge–End Collaborative Federated Learning

### 3.1. Cloud–Edge–End Federated Learning Framework

**Figure 1:**
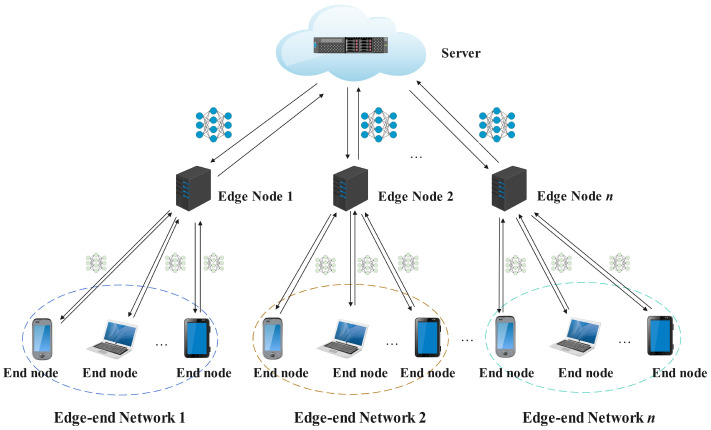
Federated learning framework for cloud–edge–end architecture.

Figure 1 illustrates the federated learning framework in a cloud–edge–end three-tier architecture. This architecture consists of End Nodes, Edge Nodes, and a central Server. It aims to achieve distributed machine learning tasks through federated learning while protecting client data privacy, especially in scenarios where client data are non-independent and identically distributed (non-IID).

The End Node layer consists of mobile and IoT devices that process data and update models locally, protecting privacy without uploading raw data to higher layers.

The Edge Node layer includes intermediate computing devices close to the data source (end nodes), possessing strong computational and storage capabilities. The main responsibilities of edge nodes include collecting local model updates from end nodes, aggregating these model parameters, and, when necessary, sending the aggregated model to the central server for further processing. Each edge node manages multiple end nodes, forming an “Edge–-end Network”. Edge nodes act as intermediaries between end devices and the central server, capable of performing preliminary model aggregation operations locally, reducing the burden on the central server and accelerating the global aggregation speed of the model.

The central Server is the core computational node in the federated learning architecture, responsible for receiving model updates from various edge nodes and performing global model aggregation and updates. The aggregated global model is sent back to each edge node and then distributed to various end nodes through edge nodes to complete the iterative training process of the model.

In cloud–edge–end three-tier architecture, the federated learning process is as follows:

Firstly, end nodes train models using local data and then send model updates to their associated edge nodes.

Secondly, edge nodes aggregate the received model updates, compute regional models, and then send them to the central server.

Thirdly, the central server performs a global aggregation of models from various edge nodes, updating the global model.

Finally, after the global model is updated, the central server sends it to edge nodes, which then distribute it to various end nodes, completing one training iteration and starting a new round of training.

The above cloud–edge–end architecture constitutes a hierarchical federated learning system, where end nodes train models using local data, edge nodes are responsible for preliminary aggregation, and the central Server performs global model updates. This architecture protects end node data privacy through local training and distributed computing. One challenge in this architecture is that end node data typically exhibits non-IID characteristics, meaning data distributions differ across end nodes. These data heterogeneities may lead to issues such as unstable model training and decreased model accuracy. To address the non-IID data situation at end nodes, we propose to improve model accuracy through data distribution similarity grouping strategies and hierarchical aggregation.

### 3.2. Edge Clustering Based on Similarity

**Figure 2:**
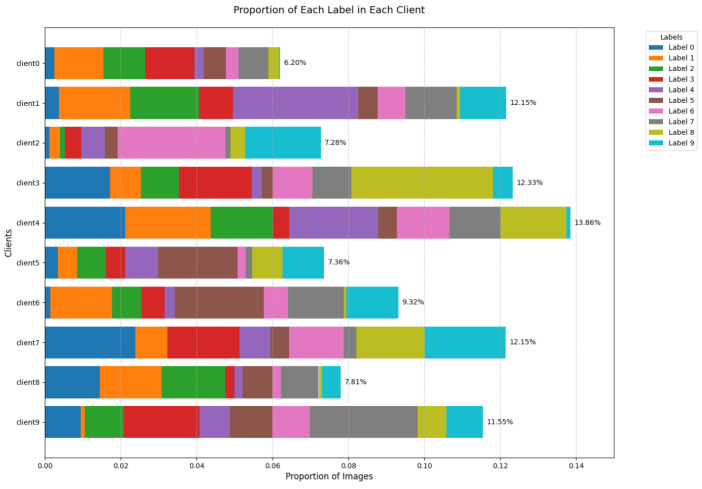
Illustration of non-IID client data in federated learning.

In cloud–edge–end architecture, the non-independent and identically distributed nature of end node data is a ubiquitous and highly challenging problem. This situation mainly manifests in label distribution skew, feature distribution differences, quantity imbalances, and inconsistent distributions of data collected at different times. Figure 2 is an illustrative diagram of client label distribution skew in federated learning based on the MNIST dataset. From this stacked bar chart, it can be observed that there is a significant label distribution skew across different clients.

Due to the different data distributions of end nodes, traditional global model aggregation methods are easily affected by extreme distributions of individual datasets, leading to insufficient model generalization ability and training instability. To address this issue, we group end nodes based on the similarity of their data distributions, partitioning end nodes with similar data distributions into the same group, which helps reduce uncertainty in model training through local data aggregation.

The first stage involves calculating the similarity of data distributions between each pair of end nodes based on their label distributions and constructing a similarity matrix *S*.

First, perform label distribution statistics. For each end node *i*, calculate the frequency of occurrence of each label *l_k_* in its dataset, computing the probability $p_{l}^{\left(i\right)}$ of label *l_k_*, as shown in the following equation:

(1) $$p_{l}^{\left(i\right)}=\frac{\left|\{(x_{i,j},y_{i,j})\in D_{i},y_{i,j}=l_{k}\}\right|}{\left|D_{i}\right|}$$

Next is the similarity calculation. For each pair of end nodes *i* and *j*, calculate the similarity $Sim(D_{i},D_{j})$ of their data distributions:

(2) $$Sim(D_{i},D_{j})=\frac{\sum_{l=1}^{L}p_{l}^{\left(i\right)}p_{l}^{\left(j\right)}}{\sqrt{\sum_{l=1}^{L}{\left(p_{l}^{\left(i\right)}\right)}^{2}}\sqrt{\sum_{l=1}^{L}{\left(p_{l}^{\left(j\right)}\right)}^{2}}}$$

Then, construct the similarity matrix. Store the similarity calculation results between all pairs of end nodes in the similarity matrix *S*, where each element *S_ij_* is the similarity between end nodes *i* and *j*.

By measuring the similarity of data distributions between clients, a foundation is provided for grouping operations. Higher similarity indicates that the data distributions of two clients are closer, and they should be grouped together to reduce the impact of non-IID on model training.

When performing edge clustering, it is essential to consider the physical connectivity between end nodes and edge nodes. The connection relationship matrix *C* between end nodes and edge nodes is defined as follows:

(3) $$C=\{c_{ij},c_{ij}\in \{0,1\},1\leq i\leq n,1\leq j\leq m\}$$

where *n* represents the number of end nodes, *m* represents the number of edge nodes, *c_ij_* = 1 indicates that there exists a physical connection between end node *i* and edge node *j*, and *c_ij_* = 0 indicates that there is no physical connection between end node *i* and edge node *j*.

By introducing connection relationship matrix *C*, the connectivity constraint is used as a constraint when constructing clusters to ensure that terminal nodes can only be clustered to edge nodes that are connected to them. The similarity matrix *S*′ with connection constraints is constructed as follows:

(4) $$S_{ij}^{\prime }=S_{ij}\cdot c_{ij}$$

Here, *c_ij_* has the same meaning as in Equation (3).

The second stage uses spectral clustering to group end nodes based on the similarity matrix *S*′, ensuring that the data distributions of end nodes within a group are as similar as possible, thereby mitigating the impact of non-IID data on federated learning.

First, construct the graph: View the similarity matrix *S*′ as the adjacency matrix of graph *G* = (*V*, *E*), where each node *v_i_* ∈ *V* represents a client, and the weight of each edge *e_ij_* ∈ *E* is the similarity *S*′*_ij_*.

Then, calculate the normalized Laplacian matrix: Compute the degree matrix ℳ of graph *G*, where ℳ is a diagonal matrix with diagonal elements and ℳ*_ii_* is the sum of weights of edges connected to node *v_i_*, i.e., $M_{ii}=\sum_{j=1}^{n}{S^{\prime }}_{ij}$.

Next, calculate the normalized Laplacian matrix *L*:

(5) $$L=M^{-\frac{1}{2}}(M-S^{\prime })M^{-\frac{1}{2}}$$

where *L* is a symmetric matrix used to capture the structural information of the graph.

Calculate eigenvectors: Perform eigenvalue decomposition on the Laplacian matrix *L* to find the eigenvectors corresponding to the *K* smallest non-zero eigenvalues. These eigenvectors form a matrix *U* with dimensions *n* × *K*. Each row of matrix *U* represents the embedding of a client in *K*-dimensional space.

Normalization: Normalize each row vector of *U* so that each vector has a norm of 1. The normalized matrix is still *U*, but each row has a length of 1.

*K*-means clustering: Perform *K*-means clustering on the row vectors of matrix *U* to partition *n* clients into *K* clusters. *K*-means clustering aims to assign clients with high similarity to the same cluster. After clustering, each cluster *G_k_* contains clients with similar data distributions. Here, the value of *K* is not greater than the number of edge nodes in the cloud–edge–end network. Finally, obtain client groupings 𝒢 = {*G*_1_, *G*_2_, …, *G_K_*}, where each *G_k_* is a set of similar clients, and these groupings can be used for subsequent federated learning model training.

The specific algorithm is shown in Algorithm 1.
**Algorithm 1** Data Distribution Similarity Clustering**Input**: End node data 𝒟*_i_*: For each end node *i*, where 𝒟*_i_* = ${\left\{x_{i,j},y_{i,j}\right\}}_{j=1}^{N_{i}}$, *x_i_*_,*j*_ represents the *j*-th data sample, and *y_i_*_,*j*_ represents the corresponding label. Number of groups *K*: The number of groups to partition the end nodes into.**Output**: End node grouping: 𝒢 = {*G*_1_, *G*_2_, …, *G_K_*}, where *G_K_* is the set of end nodes with similar data distribution in the *K*-th group1:  **for** each pair of end nodes (*i*,*j*)2:   $Sim(D_{i},D_{j})=\frac{\sum_{l=1}^{L}p_{l}^{\left(i\right)}p_{l}^{\left(j\right)}}{\sqrt{\sum_{l=1}^{L}{\left(p_{l}^{\left(i\right)}\right)}^{2}}\sqrt{\sum_{l=1}^{L}{\left(p_{l}^{\left(j\right)}\right)}^{2}}}$
3:  **end for**4:  Construct similarity matrix *S*′5: Construct graph *G* = (*V*, *E*)6:  Calculate the normalized Laplacian matrix *L* of graph *G*: $L=M^{-\frac{1}{2}}(M-S^{\prime })M^{-\frac{1}{2}}$
7:    Calculate the first *K* eigenvectors of *L* and form matrix *U*8:    Normalize each row of *U* to unit length 9:    Perform *K*-means clustering on the rows of *U* to obtain *K* clusters10:  **output** end node grouping 𝒢 = {*G*_1_, *G*_2_, …, *G_K_*}

Algorithm 1 uses spectral clustering to effectively identify clusters of end node distributions in high-dimensional data spaces by utilizing the graph structure constructed from the similarity matrix. Through dimensionality reduction using the eigenvectors of the Laplacian matrix, spectral clustering can accurately group end nodes, ultimately obtaining different groupings of end nodes based on data distribution similarity.

### 3.3. GAN-Based Data Generation

In cloud–edge–end architectures, data generated by end nodes (such as mobile devices and sensors) often exhibits non-independent and identically distributed characteristics. This means that the data distribution of each end node may be significantly different, and these data heterogeneities can affect the model accuracy in federated learning. To improve model accuracy while protecting end node data privacy, we consider generating synthetic data based on the original data from end nodes to balance data distribution.

Given that the data on each end node has non-IID characteristics, generating synthetic data with features and distributions similar to real data when there are significant differences in data distribution between different terminals is a challenging problem. To ensure the usability of the generated synthetic data and the effectiveness of privacy protection, we consider using WGAN-GP (Wasserstein Generative Adversarial Network with Gradient Penalty) to generate new synthetic datasets. By generating high-quality synthetic data, we can balance the data distribution among different end nodes, thereby improving the accuracy of the global model in federated learning.

Wasserstein Generative Adversarial Network (WGAN) [39] is an improvement over the original GAN, using Wasserstein distance as a measure between the generated distribution and the real distribution, providing stability and efficiency in training generative adversarial networks. WGAN-GP further introduces a gradient penalty term compared to WGAN, ensuring that the discriminator (called critic in WGAN) satisfies the 1-Lipschitz constraint, thereby enhancing the performance of the discriminator. The objective function of WGAN-GP is as follows:

(6) $$\min_{G}\;\max_{D}\;Ex\sim pdata[D(x)]-Ez\sim p(z)[D(G(z))]-\lambda E\hat{x}\sim p_{\hat{x}}[{\left(|\nabla_{\hat{x}}D(\hat{x}){|}_{2}-1\right)}^{2}]$$

where *G* is the generator, *D* is the discriminator (critic), *x* is a real data sample, *z* is random noise, $\hat{x}$ is an interpolation point between real and generated data, and *λ* is the weight of the gradient penalty.

#### 3.3.1. Initialize the Generator and Discriminator

The initialization of the generator *G_θ_* is as follows:

The generator’s task is to generate realistic synthetic data from a random noise vector *z*. A deep convolutional neural network (DCNN) [40] is used to construct the generator. To ensure global consistency in the generated data, a self-attention mechanism [41] is integrated into the generator, capable of capturing long-range feature dependencies and improving the quality of generated data. The parameter *θ* is initialized to small random values using a normal distribution. The weight matrices *W_Q_*, *W_K_*, *W_V_* in the self-attention layer are initialized similarly.

The initialization of the discriminator $D_{\phi }$ is as follows:

The discriminator’s task is to differentiate between real data and synthetic data generated by the generator. Similarly to the generator, the discriminator is also implemented using a deep convolutional neural network. The discriminator also integrates a self-attention mechanism to focus on important features in the data during the discrimination process. The parameter ∅ is similarly initialized to small random values. The parameters of the self-attention layer are also initialized using a normal distribution.

#### 3.3.2. Self-Attention Mechanism

In cloud–edge–end architectures, the heterogeneity of non-IID data can make it difficult for the generator to learn global features, potentially leading to the generation of low-quality data, which in turn affects the accuracy of the federated learning model. Self-attention enhances data generation by capturing feature correlations, improving synthetic data quality.

The input to the self-attention layer is a feature map *X* with dimensions $X\in {\mathbb{R}}^{C\times H\times W}$, where *C* denotes the number of channels, while *H* and *W* represent the height and width of the feature map, respectively. Query *Q*, Key *K*, and Value *V* are computed as follows:

*Q* = *W_Q_X*, where *W_Q_* is the query weight matrix.

*K* = *W_K_X*, where *W_K_* is the key weight matrix.

*V* = *W_V_X*, where *W_V_* is the value weight matrix.

The attention score *A* is calculated according to the following formula:

(7) $$A=\mathrm{softmax}(QK^{T}/\sqrt{d_{k}})$$

where *d_k_* is the dimension of the key vector. This score represents the similarity between features.

The self-attention feature map is generated as follows:

*SA*(*X*) = *AV*, i.e., a weighted sum of the value vectors *V*, generating a feature map that captures global information.

**Figure 3:**
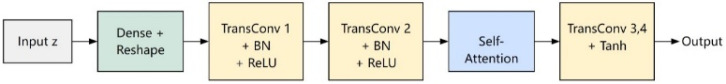
Generator structure of WGAN-GP after adding the self-attention layer.

In the WGAN-GP generator structure, the self-attention layer is inserted in the middle layers. The implementation steps for adding a self-attention layer in the generator are as follows: first, reshape the feature map into a sequence, then compute self-attention, reshape the result back to the original shape, and finally use a residual connection to add the attention output to the input. Figure 3 shows a schematic diagram of the generator structure of WGAN-GP after adding the self-attention layer.

**Figure 4:**
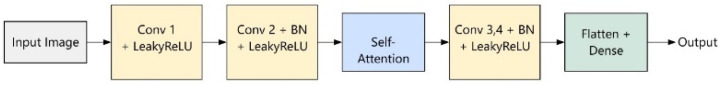
Discriminator structure of WGAN-GP after adding the self-attention layer.

Figure 4 shows a schematic diagram of the discriminator structure of WGAN-GP after adding the self-attention layer.

After adding the self-attention layer, the objective function of WGAN-GP remains unchanged, but the network structures of the generator and discriminator have changed, as shown below:

(8) $$\min_{G_{a}}\;\max_{D_{a}}\;Ex\sim pdata[D_{a}(x)]-Ez\sim p(z)[D_{a}(G_{a}(z))]-\lambda E\hat{x}\sim p_{\hat{x}}[{\left(|\nabla_{\hat{x}}D_{a}(\hat{x}){|}_{2}-1\right)}^{2}]$$

where *G_a_* and *D_a_* represent the generator and discriminator with added attention mechanisms, respectively.

Adding a self-attention mechanism to the WGAN-GP generator helps capture dependencies between distant features, making the generated data more consistent and realistic. Similarly, adding a self-attention mechanism to the WGAN-GP discriminator helps the discriminator better distinguish between real and fake data, improving the model’s discriminative ability.

#### 3.3.3. Training Loop

The generator update steps of WGAN-GP are as follows:

Step 1: Noise vector sampling: Randomly sample a batch of noise vectors $\left\{z_{1},z_{2},\ldots ,z_{m}\right\}$ from the latent space *z*. These noise vectors are the input to the generator.

Step 2: Generate fake data: Use the generator *G_θ_* to compute fake data $\hat{x}=G_{\theta }(z)$. At this point, the generator uses its network structure and self-attention mechanism to generate synthetic data with a distribution similar to real data from the noise.

Step 3: Apply self-attention mechanism: Within the generator, the self-attention mechanism performs feature extraction on the generated data, focusing on global dependencies to ensure the consistency and authenticity of the generated data.

Step 4: Calculate generator loss: The generator’s objective is to deceive the discriminator, making it believe that the generated data are real. The generator loss function is defined as the expected value of the discriminator’s output for generated data $\hat{x}$. Specifically, it is formulated as follows:

(9) $$L_{G}=-\frac{1}{m}\sum_{i=1}^{m}D_{\phi }({\hat{x}}_{i})$$

where *m* represents the batch size, which refers to the number of fake data samples generated by the generator during each training iteration, $D_{\phi }({\hat{x}}_{i})$ denotes the discriminator’s prediction for the input ${\hat{x}}_{i}$, outputting a scalar that indicates the probability of the data being real.

Calculate the gradient of the generator loss $\nabla_{\theta }L_{G}$ through backpropagation and update the generator parameters *θ* using the Adam optimizer.

The discriminator update steps for WGAN-GP are as follows:

Step 1: Real data sampling: Sample real data {*x*_1_, *x*_2_, …, *x_m_*} from the client’s original dataset.

Step 2: Apply self-attention mechanism: Apply the self-attention mechanism in the discriminator. Through this mechanism, the discriminator focuses on important areas and features in the data, enabling more accurate distinction between real and generated data.

Step 3: Calculate discriminator loss: The discriminator’s objective is to correctly distinguish between real data and generated data. The discriminator loss function is defined as follows:

(10) $$L_{D}=-\frac{1}{m}\sum_{i=1}^{m}D_{\phi }(x_{i})+\frac{1}{m}\sum_{i=1}^{m}D_{\phi }({\hat{x}}_{i})+\lambda \cdot \mathrm{GP}(x_{i},{\hat{x}}_{i})$$

where $\mathrm{GP}(x_{i},{\hat{x}}_{i})$ is the gradient penalty term, used to ensure that the discriminator satisfies the Lipschitz continuity condition.

Step 4: Backpropagation and update: Calculate the gradient of the discriminator loss $\nabla_{\phi }L_{D}$ and update the discriminator parameters *ϕ* ∅ using the Adam optimizer.

The gradient penalty is calculated in two steps. First is the interpolation data calculation. To calculate the gradient penalty term, we first need to linearly interpolate between real data *x* and generated data $\hat{x}$ to create new data $\tilde{x}$, i.e.,:

(11) $$\tilde{x}=\epsilon x+(1-\epsilon )\hat{x}$$

where *ϵ* is a random number from a uniform distribution ϵ~Uniform(0,1).

Second is the gradient penalty calculation: Calculate the gradient of the discriminator on the interpolated data $\tilde{x}$, $\nabla_{\tilde{x}}D_{\phi }(\tilde{x})$, and incorporate it into the discriminator loss:

(12) $$\mathrm{GP}(x_{i},{\hat{x}}_{i})={\left(\|\nabla_{\tilde{x}}D_{\phi }(\tilde{x}){\|}_{2}-1\right)}^{2}$$

The gradient penalty term effectively controls the gradient of the discriminator, thereby enhancing the stability of the WGAN-GP model.

The algorithm for data generation based on WGAN-GP is shown in Algorithm 2.
**Algorithm 2** Data Generation Based on WGAN-GP**Input**: Original dataset *D* = {*D*_1_, *D*_2_, …, *D_n_*}, where *D_i_* represents the dataset of the *i*-th client, *n* is the number of clients. Latent noise vector *z*, learning rate *η_G_* for generator, learning rate *η_D_* for discriminator, gradient penalty coefficient *λ*, number of iterations *T*, self-attention mechanism parameters 𝒜.**Output**: $\mathrm{Generated}\;\mathrm{synthetic}\;\mathrm{dataset}\;\hat{D}=\{{\hat{D}}_{1},{\hat{D}}_{2},\ldots ,{\hat{D}}_{n}\}$$,\;\mathrm{where}\;{\hat{D}}_{i}$ corresponds to the synthetic data generated for the *i*-th client.1: Initialize generator *G_θ_* and discriminator *D_ϕ__∅_* with parameters *θ* and *ϕ*, respectively 2: Integrate self-attention mechanism in generator and discriminator, calculate attention scores as follows: $A=\mathrm{softmax}(QK^{T}/\sqrt{d_{k}})$
3: **for each epoch**
4:   Sample a batch of noise vectors from latent space $z\;\{z_{1},z_{2},\ldots ,z_{m}\}$
5:   Compute $\hat{x}=G_{\theta }(z)$ using the generator6:   Apply self-attention mechanism on feature maps in the generator7:   Calculate generator loss $L_{G}=-\frac{1}{m}\sum_{i=1}^{m}D_{\phi }({\hat{x}}_{i})$
8:   Compute gradient $\nabla_{\theta }L_{G}$ and update generator parameters *θ* using Adam optimizer9:   Sample a batch of real data from original dataset *D_i_*
10:    Apply self-attention mechanism on feature maps in the discriminator11:    Calculate discriminator loss $L_{D}=-\frac{1}{m}\sum_{i=1}^{m}D_{\phi }(x_{i})+\frac{1}{m}\sum_{i=1}^{m}D_{\phi }({\hat{x}}_{i})+\lambda \cdot \mathrm{GP}(x_{i},{\hat{x}}_{i})$
12:    Compute gradient $\nabla_{\phi }L_{D}$ and update discriminator parameters *ϕ* using Adam optimizer 13:    Calculate $\tilde{x}=\epsilon x+(1-\epsilon )\hat{x}$   //ϵ~Uniform(0,1)
14:    Compute $\nabla_{\tilde{x}}D_{\phi }(\tilde{x})$ and incorporate it into discriminator loss15:  **end for**16: **output** synthetic datasets ${\hat{D}}_{i}$ generated for each client 

The WGAN-GP framework based on the attention mechanism proposed in this paper ensures the consistency of the generated data with the real data through a multi-level mechanism. At the feature level, the attention mechanism can effectively capture the global dependencies of the data and ensure that the generated data maintains the key features of the original data. Meanwhile, the gradient penalty term prevents excessive distortion of the generated data by limiting the gradient paradigm of the discriminator. The introduction of Wasserstein distance provides a more stable distribution matching mechanism. At the distribution level, we ensure that the generated data maintains the category balance with the original data through label distribution alignment, and the grouping mechanism of the edge network helps maintain the local data distribution characteristics. In addition, the adaptive training strategy can dynamically adjust the generation ratio according to the data quality, which further improves the reliability of the generated data. However, the method also has some potential limitations. First, the quality of generated data may be affected by the original data. Second, in terms of computational resources, generating high-quality data requires more training rounds, and the introduction of the attention mechanism increases the computational complexity, which may face a performance bottleneck on resource-constrained end devices.

### 3.4. Privacy-Preserving Federated Learning

Considering the non-IID situation of end node data, we adopt a weighted aggregation strategy that combines dataset size and data quality to ensure the reasonable evaluation of each end node’s model contribution and reduce bias. To address the typically unstable updates of models in non-IID data scenarios, we introduce an adaptive momentum factor to smooth edge model updates, reducing the impact of model update jitter on model performance. Throughout the federated learning process, the privacy of end node data is effectively protected by introducing differential privacy noise before uploading models from end nodes. The experiment is fine-tuned by introducing calibration coefficients in global aggregation to ensure better convergence of the global model when facing non-IID data.

#### 3.4.1. Balanced Processing of Non-IID Data at End Nodes

Considering the non-IID situation, data from different end nodes may have different distributions, which will lead to significant differences in the parameters of their trained models. To reduce the impact of data distribution differences, data resampling and loss function weighting strategies are introduced.

Through resampling, the local data distribution on each end node is made closer to the global data distribution, thereby reducing model bias caused by imbalanced data distribution.

Assume the global dataset’s class distribution is $P_{\mathrm{global}}=[p_{1},p_{2},\ldots ,p_{C}]$, where *p_c_* represents the proportion of class *c* in the global data, and *C* is the total number of classes. The class distribution of the local dataset for each end node *i* is $P_{i}=[p_{i,1},p_{i,2},\ldots ,p_{i,C}]$.

Define a balance factor *α* ∈ [0,1], which is used to control the intensity of resampling. The role of the balance factor is to make the resampling ratio closer to 1, i.e., to enhance data while maintaining certain distribution differences, rather than completely eliminating imbalances. The advantage of this approach is that it allows a certain degree of data imbalance, which preserves the original distribution characteristics of the dataset while reducing information loss caused by complete data resampling.

The resampling ratio ${\hat{r}}_{i,c}$ for each class *c* in the local data are calculated as follows:

(13) $${\hat{r}}_{i,c}=\alpha \cdot \frac{p_{c}}{p_{i,c}}+(1-\alpha )\cdot 1$$

where (1 − *α*)∙1 represents the tendency towards complete balance in the dataset, where 1 multiplied by (1 − *α*) signifies the “baseline” ratio for resampling. This baseline assumes that when the resampling ratio is 1, the data proportions across all categories are perfectly balanced. This formulation embodies the core principle in addressing data imbalance: maintaining a degree of imbalance while preventing the data distribution from deviating entirely from a balanced state.

Resample the local dataset $D_{i}$ of end node *i* according to the ratio ${\hat{r}}_{i,c}$ to generate a new training set ${\hat{D}}_{i}$ as follows:

(14) $${\hat{D}}_{i}=\mathrm{Resample}(D_{i},{\hat{r}}_{i})$$

where ${\hat{r}}_{i}=[{\hat{r}}_{i,1},{\hat{r}}_{i,2},\ldots ,{\hat{r}}_{i,C}]$.

The resampling ratio ${\hat{r}}_{i,c}$ ensures that the local training data distribution is closer to the global distribution. If a certain class is underrepresented in the local data, its samples can be increased through repeated sampling; conversely, if a certain class is overrepresented in the local data, its samples are reduced.

To better address the non-IID data aresue at end nodes, the loss function is weighted during the model training process to make the gradients contributed by different classes of data relatively balanced in training, thereby reducing the model’s bias towards classes with larger data distributions.

Assume that each end node *i* uses a loss function $L_{i}(\theta )$ during training, where *θ* is the model’s parameters. To weigh the loss function, first calculate the imbalance penalty factor *γ_i_*_,*c*_ for class *c*:

(15) $$\gamma_{i,c}=\frac{p_{c}}{p_{i,c}}-1$$

where *p_c_* ∈ **P**_global_, *p_i_*_,*c*_ ∈ **P***_i_*.

Define the weighting factor for each class *c* as ${\hat{w}}_{i,c}$, calculated as follows:

(16) $${\hat{w}}_{i,c}=\exp(\beta \cdot \gamma_{i,c})$$

where *β* is a parameter controlling the penalty intensity.

Construct the weighted loss function $L_{i}^{\mathrm{weighted}}(\theta )$ based on the weighting factor ${\hat{w}}_{i,c}$ as follows:

(17) $$L_{i}^{\mathrm{weighted}}(\theta )=\sum_{c=1}^{C}{\hat{w}}_{i,c}\cdot L_{i,c}(\theta )$$

where $L_{i,c}(\theta )$ is the loss function for class *c*.

#### 3.4.2. Local Model Training at End Nodes

Each end node trains the model using its local dataset combined with data generated based on WGAN-GP. In each round of the federated learning process, edge nodes obtain the latest global model $M_{\mathrm{global}}^{\left(t-1\right)}$ from the central server and distributes it to the end nodes within their subnetwork, using it as the initial model for local training. This step ensures that all nodes have a consistent starting point for their models at the beginning of each round of training. Here, *t* represents the global communication round in federated learning.

End node *i* first obtains the global model from the previous round from the edge node and initializes it as the local model $M_{i}^{\left(t,0\right)}$:

(18) $$M_{i}^{\left(t,0\right)}=M_{\mathrm{global}}^{\left(t-1\right)}$$

Then, according to the resampling ratio ${\hat{r}}_{i,c}$ in Equation (15), the local dataset *D_i_* is resampled to obtain the resampled dataset ${\hat{D}}_{i}$.

To better protect the privacy of local data at end nodes, we consider adding differential privacy noise to the model parameters of end nodes.

First, clip the model gradient $\nabla L_{i}^{\mathrm{weighted}}(M_{i}^{\left(t,e\right)},{\hat{D}}_{i})$ of end node *i*:

(19) $$\nabla {\tilde{L}}_{i}^{\mathrm{weighted}}(M_{i}^{\left(t,e\right)},{\hat{D}}_{i})=\frac{\nabla L_{i}^{\mathrm{weighted}}(M_{i}^{\left(t,e\right)},{\hat{D}}_{i})}{\max\left(1,\frac{\|\nabla L_{i}^{\mathrm{weighted}}(M_{i}^{\left(t,e\right)},{\hat{D}}_{i}){\|}_{2}}{C}\right)}$$

where $\|\nabla L_{i}^{\mathrm{weighted}}(M_{i}^{\left(t,e\right)},{\hat{D}}_{i}){\|}_{2}$ represents the *L*_2_-norm of the gradient, which is the Euclidean length of the gradient vector. *C* is the threshold hyperparameter for gradient clipping, used to set the upper limit of the gradient norm, which determines the maximum allowed value of the gradient. The $\max\left(1,\frac{\|\nabla L_{i}^{\mathrm{weighted}}(M_{i}^{\left(t,e\right)},{\hat{D}}_{i}){\|}_{2}}{C}\right)$ function represents taking the larger of two values, one of which is 1, and the other is the ratio of the gradient norm to the threshold *C*. In this way, when the gradient norm exceeds *C*, the gradient will be clipped; otherwise, the gradient remains unchanged.

Gradient clipping [42] is a method used to control the magnitude of gradient values, with the main purpose of preventing gradient explosion or vanishing gradient problems. In federated learning, gradient explosion can lead to unstable model parameter updates, thereby affecting the convergence of the model. By clipping the gradient, the maximum value of the gradient can be limited, making the gradient update process more stable.

The specific process of gradient clipping is as follows:

When the *L*_2_-norm of the gradient $\|\nabla L_{i}^{\mathrm{weighted}}(M_{i}^{\left(t,e\right)},{\hat{D}}_{i}){\|}_{2}$ is less than or equal to *C*, the value of the max function is 1, and the gradient remains unchanged:

(20) $$\nabla {\tilde{L}}_{i}^{\mathrm{weighted}}(M_{i}^{\left(t,e\right)},{\hat{D}}_{i})=\nabla L_{i}^{\mathrm{weighted}}(M_{i}^{\left(t,e\right)},{\hat{D}}_{i})$$

When the *L*_2_-norm of the gradient $\|\nabla L_{i}^{\mathrm{weighted}}(M_{i}^{\left(t,e\right)},{\hat{D}}_{i}){\|}_{2}$ is greater than *C*, the gradient is scaled so that the *L*_2_-norm of the clipped gradient $\nabla {\tilde{L}}_{i}^{\mathrm{weighted}}(M_{i}^{\left(t,e\right)},{\hat{D}}_{i})$ equals *C*:

(21) $$\nabla {\tilde{L}}_{i}^{\mathrm{weighted}}(M_{i}^{\left(t,e\right)},{\hat{D}}_{i})=\frac{C}{\|\nabla L_{i}^{\mathrm{weighted}}(M_{i}^{\left(t,e\right)},{\hat{D}}_{i}){\|}_{2}}\cdot \nabla L_{i}^{\mathrm{weighted}}(M_{i}^{\left(t,e\right)},{\hat{D}}_{i})$$

Next, Gaussian noise is added to the clipped gradient $\nabla {\tilde{L}}_{i}^{\mathrm{weighted}}(M_{i}^{\left(t,e\right)},{\hat{D}}_{i})$ to implement differential privacy:

(22) $$\nabla {{\tilde{L}}_{i}}^{\prime \mathrm{weighted}}(M_{i}^{\left(t,e\right)},{\hat{D}}_{i})=\nabla {\tilde{L}}_{i}^{\mathrm{weighted}}(M_{i}^{\left(t,e\right)},{\hat{D}}_{i})+N(0,\sigma^{2}C^{2}I)$$

where $N(0,\sigma^{2}C^{2}I)$ represents Gaussian noise with mean 0 and variance $\sigma^{2}C^{2}$, while *I* is the identity matrix. This method enhances data privacy by introducing randomness into the training process. *σ*^2^ represents the variance coefficient of the noise, which is related to the privacy budget parameter. By controlling the value of *σ*, a balance between model accuracy and privacy is achieved.

Each end node *i* performs *E* iterations of gradient descent updates on the resampled dataset ${\hat{D}}_{i}$ to obtain the local model for the end node in round *t*:

(23) $$M_{i}^{\left(t,e+1\right)}=M_{i}^{\left(t,e\right)}-\eta \cdot \nabla {{\tilde{L}}_{i}}^{\prime \mathrm{weighted}}(M_{i}^{\left(t,e\right)},{\hat{D}}_{i})$$

where *e* represents the number of local training iterations within each global communication round *t*. That is, in each round *t*, end node *i* performs *E* iterations of training on the resampled dataset ${\hat{D}}_{i}$.

Finally, in the model parameter submission stage to the edge node: After adding differential privacy noise, each end node *i* submits the noisy model parameters ${\tilde{M}}_{i}^{\left(t,e+1\right)}$ to the edge node of its subnetwork for subnetwork model aggregation.

The algorithm for the local training phase of end nodes is shown in Algorithm 3.
**Algorithm 3** Local Training of Endpoints**Input**: End node data 𝒟*_i_*, global model parameters $W_{i}^{\left(0\right)}$, learning rate *η*, batch size *B*, resampling ratio ${\hat{r}}_{i,c}$, differential privacy noise parameter *σ*, number of training rounds *T***Output**: $\mathrm{Updated}\;\mathrm{local}\;\mathrm{model}\;\mathrm{parameters}\;{\tilde{M}}_{i}^{\left(t,e+1\right)}$1: Initialize model parameters $M_{i}^{\left(t,0\right)}$
2: Set hyperparameters3: Perform data resampling according to resampling ratio ${\hat{r}}_{i,c}$: ${\hat{D}}_{i}=\mathrm{Resample}(D_{i},{\hat{r}}_{i})$4: **for each epoch**5:    Gradient clipping for end node *i*’s model:     $\nabla {\tilde{L}}_{i}^{\mathrm{weighted}}(M_{i}^{\left(t,e\right)},{\hat{D}}_{i})=\frac{\nabla L_{i}^{\mathrm{weighted}}(M_{i}^{\left(t,e\right)},{\hat{D}}_{i})}{\max\left(1,\frac{\|\nabla L_{i}^{\mathrm{weighted}}(M_{i}^{\left(t,e\right)},{\hat{D}}_{i}){\|}_{2}}{C}\right)}$
6:    Add Gaussian noise to clipped gradients:    $\;\;\nabla {\tilde{L}}_{i}^{\prime \mathrm{weighted}}(M_{i}^{\left(t,e\right)},{\hat{D}}_{i})=\nabla {\tilde{L}}_{i}^{\mathrm{weighted}}(M_{i}^{\left(t,e\right)},{\hat{D}}_{i})+N(0,\sigma^{2}C^{2}I)$
7:    Update model parameters using gradients with differential privacy:     $\;\;M_{i}^{\left(t,e+1\right)}=M_{i}^{\left(t,e\right)}-\eta \cdot \nabla {{\tilde{L}}_{i}}^{\prime \mathrm{weighted}}(M_{i}^{\left(t,e\right)},{\hat{D}}_{i})$
8: **end for**
9: **output** updated local model parameters ${\tilde{M}}_{i}^{\left(t,e+1\right)}$


#### 3.4.3. Edge Node Subnetwork Model Aggregation

In edge computing scenarios, local models ${\tilde{M}}_{i}^{\left(t,e+1\right)}$ generated by each end node *i* are uploaded to their respective edge node *j* for aggregation. Due to differences in factors such as dataset size and quality among end nodes, simply averaging all local models directly may cause low-quality data from certain nodes to have an excessively negative impact on the aggregated model. Therefore, we consider introducing a weighted aggregation strategy at edge nodes, assigning weights to each end node to ensure that the aggregation results better reflect the diversity and quality of the datasets.

Suppose an edge node *j* has multiple end nodes $I_{j}=\{i_{1},i_{2},\ldots ,i_{k}\}$ in its subnetwork, and these end nodes have uploaded their locally trained models ${\tilde{M}}_{i_{1}}^{\left(t,e+1\right)},{\tilde{M}}_{i_{2}}^{\left(t,e+1\right)},\ldots ,{\tilde{M}}_{i_{k}}^{\left(t,e+1\right)}$. The aggregated model $M_{j}^{\left(t\right)}$ of the edge node is generated through the following weighted aggregation formula:

(24) $$M_{j}^{\left(t\right)}=\sum_{i\in I_{j}}l_{i}\cdot {\tilde{M}}_{i}^{\left(t,e+1\right)}$$

The weight factor $l_{i}$ reflects the importance of each end node’s model, calculated according to the following formula:

(25) $$l_{i}=\frac{\left|D_{i}|\cdot \mathrm{Quality}(D_{i}\right)}{\sum_{k\in I_{j}}|D_{k}|\cdot \mathrm{Quality}(D_{k})}$$

where $\left|D_{i}\right|$ represents the size of the local dataset of end node *i*, i.e., the number of data samples. $\mathrm{Quality}(D_{i})$ represents the quality assessment value of end node *i*’s dataset, reflecting the effectiveness of the dataset and its contribution to model training, usually evaluated through model performance on the local validation set, data noise level, degree of deviation from data distribution, etc. The denominator in the formula ensures that the sum of weights $l_{i}$ is 1, ensuring that the weighted aggregation result does not bias towards any specific node but reflects the comprehensive contribution of all nodes.

$\mathrm{Quality}(D_{i})$ is defined as follows:

(26) $$\mathrm{Quality}(D_{i})=a\cdot {\mathrm{Acc}}_{i}+b\cdot \frac{1}{{\mathrm{Noise}}_{i}}+c\cdot \frac{1}{{\mathrm{Dist}}_{i}}$$

where Acc*_i_* represents the accuracy metric of the model on the local validation set of end node *i*. Noise*_i_* represents the noise level in node *i*’s dataset, which can be estimated by the proportion of noisy data. Dist*_i_* represents the degree of deviation between node *i*’s data distribution and the global data distribution. *a*, *b*, and *c* are weighting factors used to adjust the influence of each factor on the overall quality assessment.

To smooth the model update process, we introduce an adaptive momentum update mechanism.

After edge node *j* generates the preliminary aggregated model $M_{j}^{\left(t\right)}$, a momentum factor *ω* is introduced to smooth the model update:

(27) $$M_{j}^{\left(t+1\right)}=\omega \cdot M_{j}^{\left(t\right)}+(1-\omega )\cdot M_{j}^{\mathrm{agg}(t)}$$

where $M_{j}^{\left(t\right)}$ represents the model of edge node *j* in round *t*, $M_{j}^{\mathrm{agg}(t)}$ represents the model of edge node *j* obtained through weighted aggregation in round *t*.

By introducing the momentum factor *ω*, the update process becomes smoother, avoiding excessive update magnitudes when the model faces drastic fluctuations in non-IID data, thereby maintaining model stability. Since each round’s model update depends on the model from the previous round, this recursive smoothing update can, to some extent, mitigate the model instability brought about by non-IID data, ensuring the convergence of the global model.

After weighted aggregation and adaptive momentum update, the edge node uploads the final aggregated model $M_{j}^{\left(t+1\right)}$ to the central server for global aggregation.

The edge node adopts a weighted aggregation strategy, considering the quality and quantity of data from different end nodes, mitigating model bias caused by non-IID data. At the same time, the adaptive update strategy makes model updates smoother, helping to improve the stability of the aggregated model. In cloud–edge–end architecture, edge nodes serve as a buffer, performing preliminary aggregation of local model parameters uploaded from end nodes, which on one hand reduces the computational burden on the central server, and to some extent hides the data characteristics of individual end nodes.

The algorithm for edge node subnetwork aggregation is shown in Algorithm 4.
**Algorithm 4** Edge Node Sub-network Aggregation**Input**: End nodes $I_{j}=\{i_{1},i_{2},\ldots ,i_{k}\}$$,\;\mathrm{collection}\;\mathrm{of}\;\mathrm{end}\;\mathrm{node}\;\mathrm{local}\;\mathrm{model}\;\mathrm{parameters}\;{\left\{{\tilde{M}}_{i_{j}}^{\left(t\right)}\right\}}_{j=1}^{k}$, end node datasets 𝒟*_i_*, momentum factor *ω***Output**: Aggregated edge node sub-network model parameters $M_{j}^{\left(t+1\right)}$1: Initialize aggregated model parameters $M_{j}^{\left(0\right)}$
2: Initialize momentum factor *ω*3: Calculate weight factors $l_{i}$: $l_{i}=\frac{\left|D_{i}|\cdot \mathrm{Quality}(D_{i}\right)}{\sum_{k\in I_{j}}|D_{k}|\cdot \mathrm{Quality}(D_{k})}$
4: **for each epoch**5:     Perform weighted model aggregation for edge nodes based on weight factors:
$\;\;\;\;\;M_{j}^{\left(t\right)}=\sum_{i\in I_{j}}l_{i}\cdot {\tilde{M}}_{i}^{\left(t,e+1\right)}$
6:     Smooth update of model parameters using momentum factor:      $M_{j}^{\left(t+1\right)}=\omega \cdot M_{j}^{\left(t\right)}+(1-\omega )\cdot M_{j}^{\mathrm{agg}(t)}$
7: **end for**
8: Send updated model $M_{j}^{\left(t+1\right)}$ to the central server9: **output** updated local model parameters
$M_{j}^{\left(t+1\right)}$


#### 3.4.4. Global Model Aggregation

In the global aggregation process of federated learning in cloud–edge–end architecture, to address the impact of non-IID data from end nodes on model accuracy and stability, we propose the following improvement strategies: alignment calibration before global model aggregation and global model aggregation based on weighted global aggregation strategy.

Before aggregation, the central server first performs alignment calibration on the local models uploaded from various edge nodes to mitigate model parameter differences caused by non-IID data.

Alignment calibration includes two steps: normalization and feature alignment. First, normalization is performed with the main goal of eliminating scale differences in local model parameters uploaded from different edge nodes. Due to inconsistent data distribution across edge nodes, the model parameters they train may have different numerical ranges, and directly aggregating these models may lead to instability in the global model.

For the local model parameters $M_{j}^{\left(t\right)}$ of each edge node *j*, perform the following normalization:

(28) $${{\hat{M}}^{\prime }}_{j}^{\left(t\right)}=\frac{M_{j}^{\left(t\right)}-\mu_{j}}{\sigma_{j}}$$

where *μ_j_* and *σ_j_* are the mean and standard deviation of the model parameters $M_{j}^{\left(t\right)}$, respectively, and ${{\hat{M}}^{\prime }}_{j}^{\left(t\right)}$ are the normalized model parameters. Through normalization, the scale differences between model parameters of different nodes can be reduced, especially adjusting for shifts caused by non-IID data.

Next, perform feature alignment on the normalized model parameters ${{\hat{M}}^{\prime }}_{j}^{\left(t\right)}$, with the purpose of reducing the deviation between the model parameters uploaded by each edge node and the global model parameters from the previous round in each aggregation process. Feature alignment on the normalized model parameters ${{\hat{M}}^{\prime }}_{j}^{\left(t\right)}$ further ensures that the parameters of each local model remain relatively consistent with the global model from the previous round, mitigating fluctuations in model parameters.

Assuming the global model parameters from the previous round are $M^{\mathrm{global}(t-1)}$, and the normalized model parameters of the current edge node *j* are ${{\hat{M}}^{\prime }}_{j}^{\left(t\right)}$, the feature alignment operation is as follows:

(29) $${{\hat{M}}^{\prime \prime }}_{j}^{\left(t\right)}={{\hat{M}}^{\prime }}_{j}^{\left(t\right)}+\zeta (M^{\mathrm{global}(t-1)}-{{\hat{M}}^{\prime }}_{j}^{\left(t\right)})$$

where ${{\hat{M}}^{\prime \prime }}_{j}^{\left(t\right)}$ are the model parameters after feature alignment, ζ ∈ [0,1] is the alignment coefficient for feature alignment used to control the degree to which the current model parameters approach the global model parameters from the previous round.

In the above alignment operation, for the normalized model parameters ${{\hat{M}}^{\prime }}_{j}^{\left(t\right)}$ of each edge node and the global model parameters from the previous round $M^{\mathrm{global}(t-1)}$, first calculate their difference $M^{\mathrm{global}(t-1)}-{{\hat{M}}^{\prime }}_{j}^{\left(t\right)}$, then use the alignment coefficient ζ for feature alignment, adjusting the model parameters to be closer to the global model parameters. Through feature alignment, the model parameters uploaded by edge nodes are not only unified in scale but also consistent in direction and value with the global model from the previous round. This alignment helps reduce parameter inconsistencies during global model aggregation, avoiding model instability caused by non-IID data.

After receiving the calibrated local models uploaded from all edge nodes, the central server performs global model aggregation. Considering the dataset size, data quality, and historical performance of different nodes, a weighted global aggregation strategy is adopted for global model updates.

The global model update formula is as follows:

(30) $$M^{\mathrm{global}(t+1)}=\sum_{j=1}^{K}w_{j}^{\left(t\right)}\cdot {{\hat{M}}^{\prime \prime }}_{j}^{\left(t\right)}$$

The global model $M^{\mathrm{global}(t+1)}$ is the weighted average of the calibrated local models ${{\hat{M}}^{\prime \prime }}_{j}^{\left(t\right)}$ uploaded by each edge node. The weight factor $w_{j}^{\left(t\right)}$ reflects the contribution of edge node *j* in round *t*, specifically defined as follows:

(31) $$w_{j}^{\left(t\right)}=\frac{N_{j}\cdot \mathrm{Quality}(D_{j})}{\sum_{j=1}^{K}N_{j}\cdot \mathrm{Quality}(D_{j})}$$

The weight factor $w_{j}^{\left(t\right)}$ is the product of the dataset size *N_j_* and data quality $\mathrm{Quality}(D_{j})$ of edge node *j*, normalized to ensure that the sum of weights for all nodes is 1. This ensures that nodes with larger data volumes and higher quality contribute more to the global model, while nodes with lower data quality or smaller data volumes contribute less.

Finally, the distribution and cyclic iteration of the aggregated global model. After completing global model aggregation, the central server distributes the updated global model to each edge node, which in turn distributes it to its affiliated end nodes. Subsequently, edge nodes and end nodes conduct the next round of local training and aggregation based on the latest global model. This process will continue to cycle until preset convergence conditions are met.

The process of global model distribution is as follows: The central server distributes the latest global model $M^{\mathrm{global}(t+1)}$ to all edge nodes *j*, which further distribute it to the end nodes within their subnetworks. All end nodes conduct the next round of local training based on this global model.

The entire federated learning process is a cyclic iteration, with each round including local training, edge aggregation, and global aggregation. As the number of iterations increases, the global model gradually converges, ultimately resulting in a global model that can generalize to all nodes. Model distribution and cyclic iteration are core operations of federated learning, continuously improving the global model through multiple rounds of training and aggregation, maintaining high accuracy and stability even in non-IID data environments.

The global model aggregation algorithm for the central server is shown in Algorithm 5.
**Algorithm 5** Central Server Global Model Aggregation**Input**: $\mathrm{Set}\;\mathrm{of}\;\mathrm{model}\;\mathrm{parameters}\;\mathrm{uploaded}\;\mathrm{by}\;\mathrm{each}\;\mathrm{edge}\;\mathrm{node}\;{\left\{M_{k}^{\left(t\right)}\right\}}_{k=1}^{K}$, where *K* is the number of edge nodes, alignment calibration coefficient ζ, weight factor *w* for global aggregation weighting**Output**: $\mathrm{Aggregated}\;\mathrm{global}\;\mathrm{model}\;\mathrm{parameters}\;M^{\mathrm{global}}$1:  Initialize$\;\mathrm{global}\;\mathrm{model}\;\mathrm{parameters}\;M^{\left(0\right)}$
2:  Set alignment calibration coefficient ζ and weight factor *w*3:  **while** global model is not converge **do**4:   **for each**
$M_{k}^{\left(t\right)}\in {\left\{M_{k}^{\left(t\right)}\right\}}_{k=1}^{K}$
5:    Perform normalization: ${{\hat{M}}^{\prime }}_{j}^{\left(t\right)}=\frac{M_{j}^{\left(t\right)}-\mu_{j}}{\sigma_{j}}$
6:    Further perform feature alignment: ${{\hat{M}}^{\prime \prime }}_{j}^{\left(t\right)}={{\hat{M}}^{\prime }}_{j}^{\left(t\right)}+\zeta (M^{\mathrm{global}(t-1)}-{{\hat{M}}^{\prime }}_{j}^{\left(t\right)})$
7:   **end for**
8:   Calculate weight factors: $w_{j}^{\left(t\right)}=\frac{N_{j}\cdot \mathrm{Quality}(D_{j})}{\sum_{j=1}^{K}N_{j}\cdot \mathrm{Quality}(D_{j})}$
9:   Global model aggregation: $M^{\mathrm{global}(t+1)}=\sum_{j=1}^{K}w_{j}^{\left(t\right)}\cdot {{\hat{M}}^{\prime \prime }}_{j}^{\left(t\right)}$
10:  **end while**11:  **output**$\;\mathrm{aggregated}\;\mathrm{global}\;\mathrm{model}\;\mathrm{parameters}\;M^{\mathrm{global}}$


## 4. Experimental Evaluation

To validate the effectiveness of the proposed cloud–edge–end collaborative privacy-preserving federated learning method, we compared it with existing methods and analyzed the experimental results. We first describe the experimental setup, datasets, and evaluation metrics, followed by experimental verification of the proposed method from different dimensions for various scenarios.

### 4.1. Experimental Setup

#### 4.1.1. Experimental Environment and Hyperparameter Setting

The simulation experiment was developed in PyCharm 17.0.10, using Python 3.9 as the programming language and PyTorch 2.3 as the deep learning framework. The hardware is ThinkPad P71, Lenovo, Beijing, China. The specific configuration is as follows: Intel^®^ Core^TM^ i7-7820HQ CPU @ 2.90 GHz, 32 G RAM, NVIDIA Quadro P5000 GPU with 16G VRAM, and 2TB HDD.

The experiment simulates a three-layer cloud–edge–end architecture, including 10 end nodes, each storing local data with non-IID characteristics. It includes three edge nodes, with subnetworks formed by end nodes and edge nodes based on the data distribution of end nodes. Each edge node in a subnetwork is responsible for collecting local model parameters uploaded by end nodes and performing local aggregation, as well as distributing global model parameters from the central server to end nodes within its subnetwork. The central server receives local models uploaded by each edge node for global aggregation and distributes the updated global model to edge nodes.

The parameter optimization process of the WGAN-GP model is as follows: determine the range of the base parameters through pre-experimentation on a small-scale dataset, use grid search to find the optimal learning rate and batch size, and determine the final configurations based on the quality of the generated samples and the training stability. In the process of differential privacy parameter tuning, the trade-off relationship between privacy budget and model performance is first analyzed, the noise scale is determined by Privacy Loss test, and the parameters are adjusted according to the privacy requirements of the actual application scenarios. Table 1 shows the key hyperparameter setting table.

#### 4.1.2. Datasets

MNIST dataset [43]: One of the most widely used benchmark datasets in machine learning and computer vision, primarily for image classification tasks. It consists of grayscale images of handwritten digits from 0 to 9, including 60,000 training samples and 10,000 test samples, with each sample being a 28 × 28 pixel handwritten digit image. The data are non-IID across end nodes.

EuroSAT dataset [44]: This dataset is a satellite remote sensing image dataset, mainly used for land use and land cover classification tasks. Based on Sentinel-2 satellite imagery, it consists of 27,000 images covering 10 different land use and cover categories, including agriculture, forest, residential areas, industrial areas, water bodies, roads, vegetation, etc. Each image is 64 × 64 pixels and contains 13 multispectral bands. 90% of the dataset is used for training, and the remaining 10% for testing.

WGAN-GP generated dataset: Synthetic data generated based on MNIST and EuroSAT datasets, with the specific generation process described in Section 3.3 of this paper, used for mixed training with original data.

**Figure 5:**
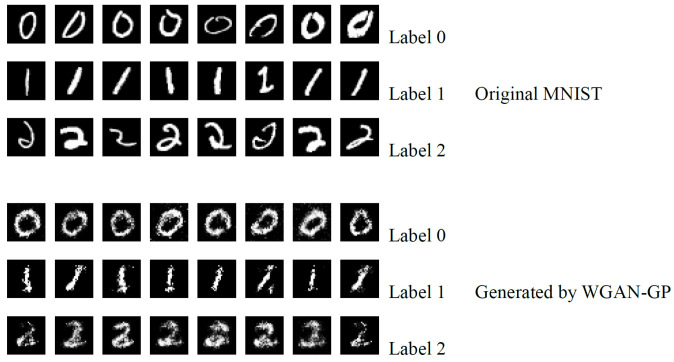
Examples of original MNIST dataset and WGAN-GP generated dataset.

Figure 5 shows examples of the original MNIST dataset and the synthetic dataset generated based on WGAN-GP.

**Figure 6:**
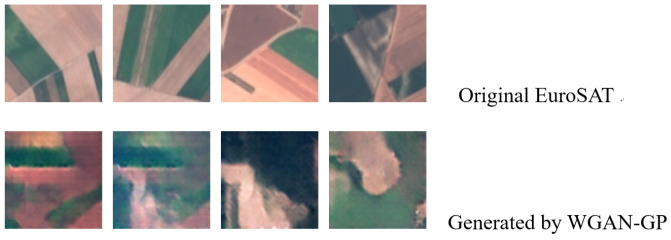
Examples of AnnualCrop label category from original EuroSAT and WGAN-GP generated dataset of the same label category.

Figure 6 shows examples of the AnnualCrop label category from the original EuroSAT and the synthetic dataset of the same label category generated based on WGAN-GP.

#### 4.1.3. Evaluation Metrics

**Accuracy**: Accuracy is an important metric for evaluating the performance of classification models, representing the proportion of correctly classified samples to the total number of samples. The formula is as follows:

(32) $$\mathrm{Accuracy}=\frac{TP+TN}{TP+TN+FP+FN}$$

where *TP* (True Positives) represents the number of samples correctly classified as positive, *TN* (True Negatives) represents the number of samples correctly classified as negative, *FP* (False Positives) represents the number of samples incorrectly classified as positive, and *FN* (False Negatives) represents the number of samples incorrectly classified as negative. Higher model accuracy indicates stronger classification ability for the given task.

**F1-Score**: F1-Score is a metric that comprehensively evaluates model precision and recall. It is the harmonic mean of precision and recall, measuring the model’s performance in predicting the positive class. The formula is as follows:

(33) $$F1\text{-}\mathrm{Score}=2\cdot \frac{\mathrm{Precision}\cdot \mathrm{Recall}}{\mathrm{Precision}+\mathrm{Recall}}$$

where $\mathrm{Precision}=\frac{TP}{TP+FP}$ represents the proportion of true positives among all samples predicted as positive. $\mathrm{Recall}=\frac{TP}{TP+FN}$ represents the proportion of correctly predicted positives among all actual positive samples. F1-Score is particularly suitable for class imbalance problems as it strikes a balance between precision and recall.

### 4.2. Experimental Results and Analysis

To better compare the effectiveness of the proposed cloud–edge–end collaborative privacy-preserving federated learning method (CEECFed), we compare it with classic federated learning methods such as FedGS [26], FedAvg [45], and FedSGD [46]. To ensure the accuracy of the experimental results, the data in this study were obtained by averaging the results from three independent runs.

#### 4.2.1. Evaluation of Model Performance Metrics

To evaluate the performance of the proposed privacy-preserving federated learning method (CEECFed) in different scenarios, we validated it through image recognition tasks using the MNIST dataset, EuroSAT dataset, and synthetic data generated based on MNIST and EuroSAT.

**Figure 7:**
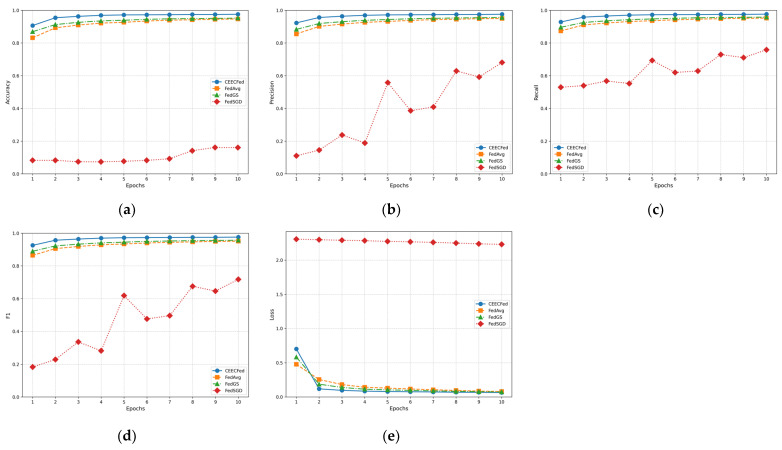
Performance of CEECFed, FedGS, FedAvg, and FedSGD based on the original MNIST dataset. (**a**) Accuracy, (**b**) Precision, (**c**) Recall, (**d**) F1-Score, (**e**) Average Loss.

Figure 7 shows the performance of CEECFed, FedGS, FedAvg, and FedSGD based on the original MNIST dataset.

**Figure 8:**
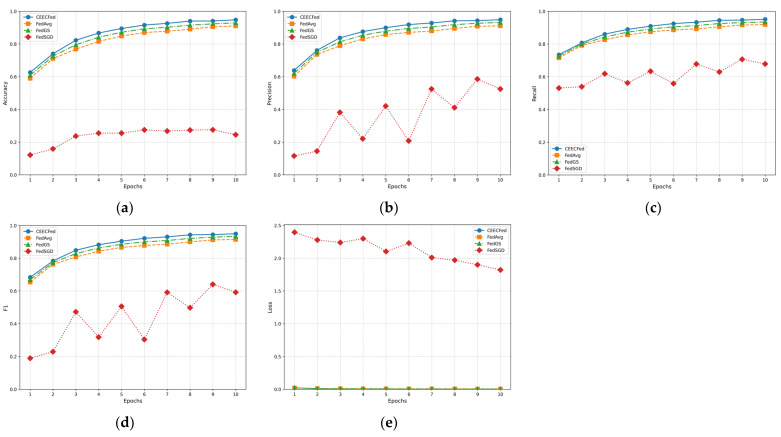
Performance of CEECFed, FedGS, FedAvg, and FedSGD based on the original EuroSAT dataset. (**a**) Accuracy, (**b**) Precision, (**c**) Recall, (**d**) F1-Score, (**e**) Average Loss.

Figure 8 shows the performance of CEECFed, FedGS, FedAvg, and FedSGD based on the original EuroSAT dataset.

In Figure 7 and Figure 8, the experimental evaluation metrics include Accuracy, Precision, Recall, F1-Score, and Average Loss.

Figure 7a and Figure 8a show the accuracy performance of CEECFed, FedGS, FedAvg, and FedSGD on the original MNIST dataset and the original EuroSAT dataset. It can be observed that throughout the training process, the CEECFed method consistently maintains the highest accuracy level compared to FedGS, FedAvg and FedSGD. The CEECFed method can effectively learn useful features in the early stages of training and stably maintain high accuracy in the subsequent training process. FedGS was a close second, with a lower final accuracy than CEECFed, but significantly better than the performance of FedAvg. FedAvg’s accuracy, while also trending upward, was slower to converge and ultimately less effective. In comparison, FedSGD has significant disadvantages in global convergence speed and generalization ability due to only one local update per round.

From Figure 7b and Figure 8b, it can be seen that in terms of precision, the CEECFed method outperforms FedGS, FedAvg and FedSGD, indicating that this method can effectively reduce the proportion of false positive samples. FedGS method’s precision is second to CEECFed, while FedSGD method’s precision is far lower than the other two methods. CEECFed’s performance in precision indicates that it can more effectively capture positive class features in the data, thereby reducing false positives. FedSGD, due to its lower update frequency, shows deficiencies in feature capture and stability.

According to Figure 7c and Figure 8c, in terms of recall, the CEECFed method performs better than FedGS, FedAvg, and FedSGD. FedSGD’s recall fluctuates greatly throughout the training process, showing poor performance. CEECFed’s performance in recall is consistent with its precision, further verifying its strong learning ability in complex data distributions. FedSGD performs poorly in recall, especially in the early stages of training, indicating significant deficiencies in capturing positive samples.

From Figure 7d and Figure 8d, it can be seen that due to CEECFed’s excellent performance in both precision and recall, its F1-score shows clear advantages over FedGS, FedAvg and FedSGD, indicating that this method performs excellently in balancing precision and recall. FedSGD’s F1-score performs poorly in the first few cycles, improves later, but still remains lower than the other two methods. CEECFed’s performance in F1-score indicates that it can effectively balance precision and recall in different tasks, thereby maintaining high overall performance.

In terms of the loss metric, from Figure 7e and Figure 8e, it can be seen that CEECFed decreases the fastest, indicating that CEECFed can effectively reduce errors and improve model performance in the early stages of training. FedAvg method’s loss decreases slightly slower than CEECFed and FedGS but better than FedSGD. FedSGD’s loss metric shows significant fluctuations throughout the training process and does not decrease significantly, displaying greater instability in the optimization process. CEECFed’s loss curve indicates that this method can quickly converge in the initial stage, significantly reducing training errors, thereby reaching a stable state faster.

Table 2 shows the performance comparison table of CEECFed, FedGS, FedAvg, and FedSGD on the original MNIST dataset. The values of Accuracy, Precision, Recall, and F1-Score are after 10 rounds. The values of the indicators that are optimal for several comparison methods are bolded in Table 2.

#### 4.2.2. Evaluation of the Effectiveness of WGAN-GP Generated Datasets

**Figure 9:**
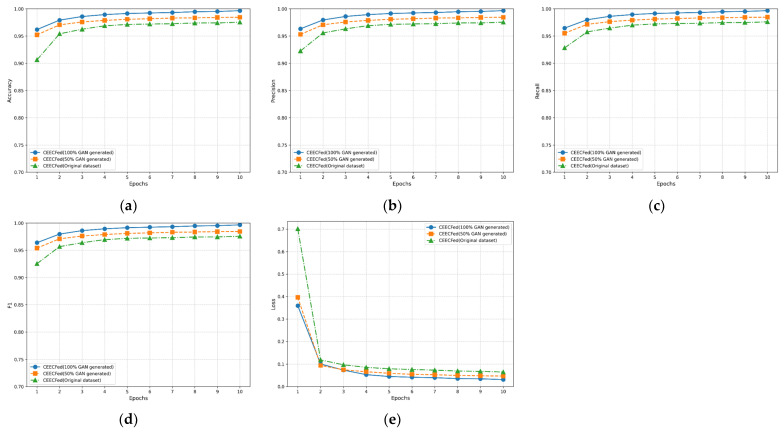
Performance of CEECFed based on original MNIST dataset and WGAN-GP generated datasets. (**a**) Accuracy, (**b**) Precision, (**c**) Recall, (**d**) F1-Score, (**e**) Average Loss.

**Figure 10:**
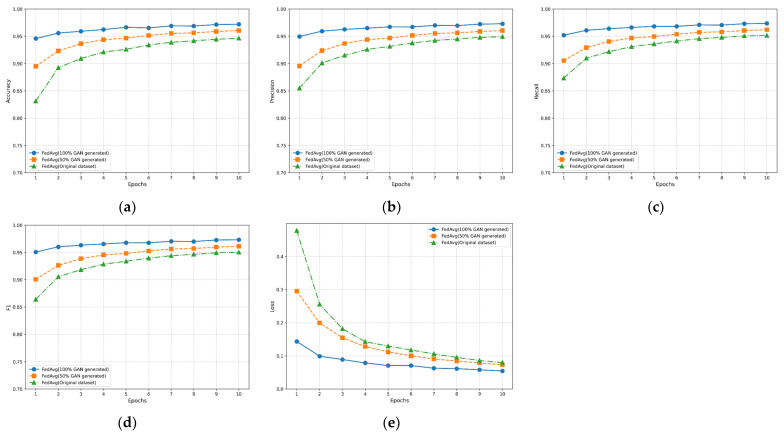
Performance of FedAvg based on original MNIST dataset and WGAN-GP generated datasets. (**a**) Accuracy, (**b**) Precision, (**c**) Recall, (**d**) F1-Score, (**e**) Average Loss.

**Figure 11:**
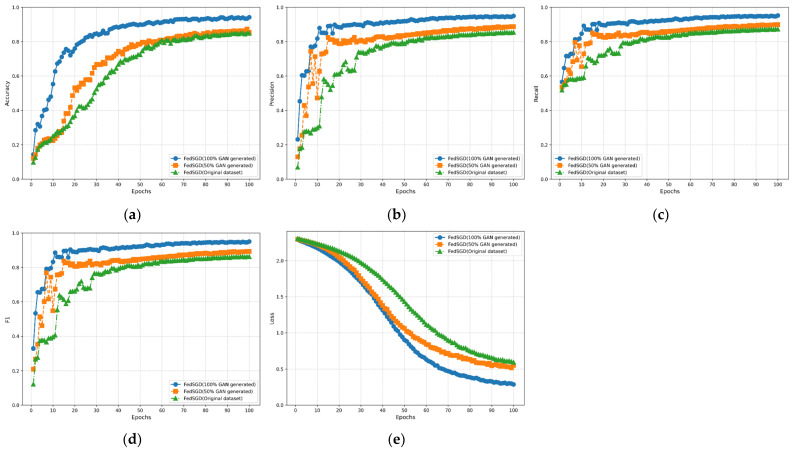
Performance of FedSGD based on original MNIST dataset and WGAN-GP generated datasets. (**a**) Accuracy, (**b**) Precision, (**c**) Recall, (**d**) F1-Score, (**e**) Average Loss.

To better evaluate the performance of the WGAN-GP method with added attention mechanism for generating datasets proposed in this paper, we validated using the original MNIST dataset (original dataset), original dataset + 50% WGAN-GP generated dataset (50% WGAN-GP generated), and 100% WGAN-GP generated dataset (100% WGAN-GP generated). Figure 9, Figure 10 and Figure 11 show the performance of CEECFed, FedAvg, and FedSGD methods on the original MNIST dataset, original dataset + 50% WGAN-GP generated data, and 100% WGAN-GP generated dataset, respectively. Experimental metrics include Accuracy, Precision, Recall, F1-Score, and Average Loss. CEECFed and FedAvg were trained for 10 rounds, while considering FedSGD’s insufficient global convergence speed and generalization ability due to only one local update per round, the experiment validated through 100 rounds of model training.

Figure 9a, Figure 10a and Figure 11a show the accuracy performance of CEECFed, FedAvg, and FedSGD methods. It can be seen that for all three methods, CEECFed, FedAvg, and FedSGD, the accuracy performance is best on the 100% WGAN-GP generated dataset, indicating that even when training entirely on generated datasets, the three methods can still maintain extremely high classification accuracy. On the combination of original dataset + partial WGAN-GP generated dataset, the accuracy of the above three methods also remains at a high level, but slightly lower than the fully WGAN-GP generated dataset. This indicates that the strategy of mixing non-IID original data with partial WGAN-GP generated data can effectively leverage the advantages of generated data, but due to differences in data distribution, it cannot fully match the features of the original data. The accuracy of the three methods on the original MNIST dataset is lower than the other two methods, indicating that in the case of non-IID data distribution, directly using original data leads to a less stable training process for the model compared to using generated data.

Figure 9b, Figure 10b and Figure 11b show the precision performance of CEECFed, FedAvg, and FedSGD methods. It is evident that the precision performance is best when using 100% WGAN-GP generated data, followed by the original dataset + partial WGAN-GP generated dataset, with the model based on the original dataset having the lowest precision. This also indicates that WGAN-GP generated data helps reduce false positives in federated learning, especially in cases of non-IID data distribution, allowing the model to better learn key features.

Figure 9c, Figure 10c and Figure 11c show the recall performance of CEECFed, FedAvg, and FedSGD methods. The experimental results show that the 100% WGAN-GP generated dataset performs most stably and efficiently, effectively detecting most positive samples. The performance on the mixed dataset is also relatively good, although slightly lower than the fully WGAN-GP generated dataset, but still better than the original dataset. This also indicates that using WGAN-GP generated datasets can significantly improve the model’s recall, especially when using fully WGAN-GP generated data, allowing the model to better cover more positive examples.

The F1-score combines the performance of precision and recall. From Figure 9d, Figure 10d and Figure 11d, it can be seen that on the 100% WGAN-GP generated dataset, the F1-score consistently remains high, indicating that the model achieves a good balance between precision and recall. The F1-score performance on the mixed dataset is better than that on the original dataset but lower than the fully WGAN-GP generated dataset. Overall, using WGAN-GP generated data can more effectively balance precision and recall, thereby achieving better overall performance in federated learning.

Figure 9e, Figure 10e and Figure 11e show the average training loss performance of CEECFed, FedAvg, and FedSGD methods. The fully WGAN-GP generated dataset shows a rapid decrease in loss in the early stages of training and maintains a low and stable level in subsequent cycles, displaying rapid convergence characteristics of the model. The mixed dataset shows slightly inferior loss performance but still displays good convergence characteristics. The original dataset shows higher loss in the early stages of training and slower convergence speed, indicating that directly training with non-IID original data may lead to an unstable model optimization process, while using WGAN-GP generated datasets can significantly reduce the model’s loss value and accelerate the model’s convergence speed.

The experimental results show that whether using 100% or partial WGAN-GP generated datasets, the performance on CEECFed, FedAvg, and FedSGD algorithms is superior to using only the original dataset. This indicates that WGAN-GP generated data can not only effectively mitigate the non-IID data distribution problem but also improve the overall performance of the model. The experimental results of this study show that by introducing WGAN-GP generated data, especially in non-IID scenarios, the performance of federated learning can be significantly improved. This provides new ideas for future research and practical applications on how to more effectively generate and utilize synthetic data to address the problem of imbalanced data distribution.

## 5. Conclusions

In cloud–edge–end scenarios, end node data may exhibit non-independent and identically distributed (non-IID) characteristics. Effectively addressing the issue of model accuracy degradation caused by non-IID data while simultaneously protecting end node data privacy presents a significant challenge. To tackle this problem, we propose a cloud–edge–end collaborative privacy-preserving federated learning method. This approach thoroughly considers the three-layer architecture of cloud–edge–end, the non-IID nature of end node data, and the typically limited computational resources, aiming to enhance model accuracy while safeguarding end node data privacy. To address the non-IID nature of end node data, we partition end nodes based on data distribution similarity and construct edge subnetworks with edge nodes for training, mitigating the negative effects of non-IID data. To counter the impact of data heterogeneity on federated learning model accuracy caused by non-IID distributions, we employ WGAN-GP with an added attention mechanism to generate high-quality synthetic data based on the original datasets from end nodes. This approach not only effectively reduces the adverse impact of non-IID data on global model accuracy but also contributes to better protection of end node data privacy. To address the significant differences in model parameters trained by different end nodes due to non-IID data, we introduce data resampling and loss function weighting strategies to reduce model bias caused by imbalanced data distribution. To further enhance end node data privacy protection, we add Gaussian noise to end node gradients, which helps resist reverse inference attacks. Experimental results based on real-world datasets demonstrate that our proposed cloud–edge–end collaborative privacy-preserving federated learning method shows significant advantages in model accuracy, F1-score, and other aspects compared to existing methods.

For future research, we plan to further optimize CEECFed, exploring its applicability to a wider range of datasets and tasks, and integrating it with other privacy protection mechanisms to enhance its practical value in real-world scenarios. Additionally, considering the potential presence of dishonest clients or edge nodes in cloud–edge–end collaborative computing architectures, we aim to investigate end node data security and privacy protection issues in scenarios involving malicious attacks.

## Figures and Tables

**Table 1:** Key hyperparameter settings.

Component	Parameter	Value
	Learning Rate	0.0002
	Batch Size	64
WGAN-GP	Gradient Penalty Weight	10
	Generator Updates/Round	5
	Attention Heads	8
Differential Privacy	Privacy Budget (ε)	3.0
Edge Clustering	Similarity Threshold	0.85
Model Training	Local Epochs	50
	Global Rounds	10

**Table 2:** Comparison of the performance of different methods on the original MNIST dataset.

Method	Accuracy (%)	Precision (%)	Recall (%)	F1-Score (%)
CEECFed	97.53	97.54	97.60	97.57
FedGS	95.18	95.52	95.78	95.65
FedAvg	94.62	94.91	95.16	95.03
FedSGD	16.02	68.08	75.80	71.74

## Data Availability

Data are contained within the article.
